# Age determination of the adult blow fly *Lucilia sericata* (Diptera: Calliphoridae) through quantitative pteridine fluorescence analysis

**DOI:** 10.1007/s12024-020-00295-4

**Published:** 2020-09-11

**Authors:** Ronja Estévez Dimitrov, Jens Amendt, Florian Rothweiler, Richard Zehner

**Affiliations:** 1 Institute of Legal Medicine, University Hospital Frankfurt, Goethe-University, Kennedyallee 104, 60596 Frankfurt am Main, Germany; 2 Institute of Medical Virology, University Hospital Frankfurt, Goethe-University, Paul-Ehrlich-Str.40, Frankfurt am Main, 60596 Germany

**Keywords:** Forensic entomology, Post mortem interval, Accumulated degree days, Age determination, Pteridine

## Abstract

Determination of a minimal postmortem interval via age estimation of necrophagous diptera has been restricted to the juvenile stages and the time until emergence of the adult fly, i.e. up until 2–6 weeks depending on species and temperature. Age estimation of adult flies could extend this period by adding the age of the fly to the time needed for complete development. In this context pteridines are promising metabolites, as they accumulate in the eyes of flies with increasing age. We studied adults of the blow fly *Lucilia sericata* at constant temperatures of 16 °C and 25 °C up to an age of 25 days and estimated their pteridine levels by fluorescence spectroscopy. Age was given in accumulated degree days (ADD) across temperatures. Additionally, a mock case was set up to test the applicability of the method. Pteridine increases logarithmically with increasing ADD, but after 70–80 ADD the increase slows down and the curve approaches a maximum. Sex had a significant impact (*p* < 4.09 × 10^−6^) on pteridine fluorescence level, while body-size and head-width did not. The mock case demonstrated that a slight overestimation of the real age (in ADD) only occurred in two out of 30 samples. Age determination of *L. sericata* on the basis of pteridine levels seems to be limited to an age of about 70 ADD, but depending on the ambient temperature this could cover an extra amount of time of about 5–7 days after completion of the metamorphosis.

## Introduction

Forensic entomology investigates insects and other arthropods as a part of legal cases, where one of its most important tasks is estimating the period of time since death or placement of a body [1]. Especially in cases where there is advanced decomposition of a body, insects may be the only way to draw conclusions about the possible time of death, which is highly relevant in homicides as well as in other scenarios. In times of an ageing population in Western and other countries and an increasing number of older singles or single households in general [2, 3], finding bodies in an advanced stage of decomposition and undetected for a longer period of time, especially indoors, is becoming more common. In fact, 85% of the bodies with insect infestation examined at our institute within the last 2 years were found indoors (unpublished data). Even though the majority of these cases did not involve homicides, it is still good practice to make an accurate statement about the time of death. Knowing the date of death helps to quickly make an initial assessment of what happened.

The time between death and discovery of the body is known as the post-mortem interval (PMI). Since external circumstances can delay the arrival of necrophagous insects and their subsequent colonization of a body, the period of time that is determined by entomological investigations is therefore referred to as a *minimum* post-mortem interval (PMI_min_). One standard to determine the PMI_min_ is to identify the insect species found on the body and their state of development or age, which corresponds with the time they need to reach this state in given environmental conditions beginning with the colonization of the body [4].

The most common insects found on dead bodies are necrophagous flies and their offspring - in cases where the PMI exceeds 72 h, they are considered the most accurate tools to determine PMI_min_ [5, 6]. Blow flies (Diptera: Calliphoridae) are of special importance since they are not only omnipresent but usually also the first at the site [7].

Depending on temperature and species, blow flies develop over a period of 2–4 weeks after oviposition until the fly has completed its metamorphosis and hatched [8]. Various landmarks of development such as eggs, the growing size of larvae, their stage of development and the time of pupariation as well as the hatching of the adult fly allow conclusions about the time of oviposition and hence about the PMI_min_.

Aging of adult flies that remain at the scene on the body in question after finishing their development might open a new door of PMI_min_ estimation, especially when it comes to indoor investigations where hatched flies have trouble leaving the room due to closed conditions [6]. So far, there have been different approaches for age grading adult flies, but they are often time consuming, imprecise and of limited use [9]. The most common procedure is the estimation of age based on the stage of development of the ovaries of female flies. Studies with different species such as tsetse flies *Glossina* spp. or forensically relevant blow flies *Lucilia cuprina* [9] and *L. sericata* [10] achieved good results, but also reported numerous limitations of the method, such as interference due to protein uptake, availability of oviposition site and mating status, as well as strong temperature sensitivity and difficulties in determining the number of previous ovarian cycles. In the field of somatic changes, there are different approaches, such as the evaluation of qualitative and quantitative differences in cuticular hydrocarbons [11, 12]. Studies on the blow flies *L. sericata*, *Calliphora vicina* [13] and the mosquito *Aedes aegypti* [14] show promising results, but also a strong influence of temperature, humidity and artificial laboratory conditions on the production and presence of cuticular hydrocarbons and the reproducibility still has to be investigated. Counting bands on muscle apodemes or the layers in the cuticle as a measure of the age of young adult insects [9, 15] is a further method but is limited to the teneral stage. Temperature, and especially temperature fluctuations between night and day, showed a strong impact on layer growth. Age determination by mechanical damage of the insect investigates things like wing fray as a measure of age. Of all the methods mentioned so far, the latter is the oldest but also the most inaccurate method of age determination, as wing damage does not just increase with age, but is caused or impacted by very local and unique environmental factors such as predator attacks, or frequency of wing movement, i.e. flight activity [9, 16].

Another method of using age dependent physical changes to help determine a specimens age is to correlate the age of an adult fly with the pteridine level in its eyes, measured by fluorescence intensity [9]. Studies on blowflies and house flies *C. vicina* [8], *Musca domestica* [17] and *Chrysomya megacephala* [18] showed a clear increase of pteridine with age. While sex and temperature, in addition to age, show a clear influence on the pteridine level during fly aging [10, 17–20], diet [10, 17–19] and light conditions [17, 20] do not.

Pteridines were named after they were discovered in wing pigments (Greek “ptera”) of pierid butterflies and were observed to accumulate in the eyes of flies with increasing age [21]. They are substances derived from the pyrimidine-pyrazine ring and seem to be present in all living organisms, with insects appearing to contain them in higher concentrations than most others. They have many varied functions and roles, including being described as important co-factors in cell metabolism and as signal molecules [22], protective antioxidants [23], UV absorbing filters, pigments for protection, recognition or communication [21, 24] and even as a way of endogenous nitrogen excretion for some insects [22]. Their characteristic of absorbing UV light appears to be their most important role in compound eyes, as increased light sensitivity can be observed in flies without pteridine [25]. Pteridines show a constant and extended linear correlation between the intensity of fluorescence and the amount of substance present, and the high sensitivity of the method is suitable for even small sample sizes [21, 26].

Age determination by pteridine levels has been investigated in several insects (including many necrophagous flies), such as *Boettcherisca peregrina* [6], *C. vicina* [8], *Ch. bezziana* [19], *Ch. megacephala* [18, 27], *Cochliomyia macellaria* [27], *M. domestica* [17], *Phormia regina* [27], and *L. sericata* [10]. The latter is one of the most frequently encountered species of necrophagous flies in Western Europe [28–30].

The aim of this study was to determine whether quantitative pteridine measurement by fluorescence analysis is suitable for age estimation of the adult blow fly *Lucilia sericata* and thus useful for supporting the entomological determination of the PMI_min_. In addition, the impact of sex and size of the fly head and body on the pteridine level was examined, since the possibility of interference by these factors has been mentioned before [9, 10, 17, 26, 31].

Compared to previous studies on *L. sericata* [e.g. 10], we modified the method in the present study, analyzing a larger number of specimens per age and temperature condition, and applying accumulated degree days (ADD) for age assessment across temperatures.

## Materials and methods

### Fly stocks

A colony of *L. sericata* was established based on field catches and rearing from specimens sampled during autopsies. Adults were kept in 57 × 38 × 36 cm rearing cages, provided with sugar and water ad libitum at room temperature (20 °C ± 2 °C) and a 12:12 light dark cycle. Once a week, a piece of beef liver (~5 g) was provided as a source of protein and as an oviposition medium for maintaining the stock.

### Producing the age cohorts

24 h after oviposition on the beef liver, the newly hatched larvae were transferred on to minced meat, consisting of half beef and half pork. Plastic cups containing 40 g minced meat and about 40 larvae were placed in cubic plastic containers (10.5 × 10.5 × 8 cm). The bottom of the plastic container was covered with wood shavings, which served as medium for pupariation. The container was closed with a lid that had tiny air holes to allow oxygen to enter while preventing the larvae from escaping during the migration phase. The containers were incubated at 16° or 25 °C untill pupariation.

**Fig. 1:**
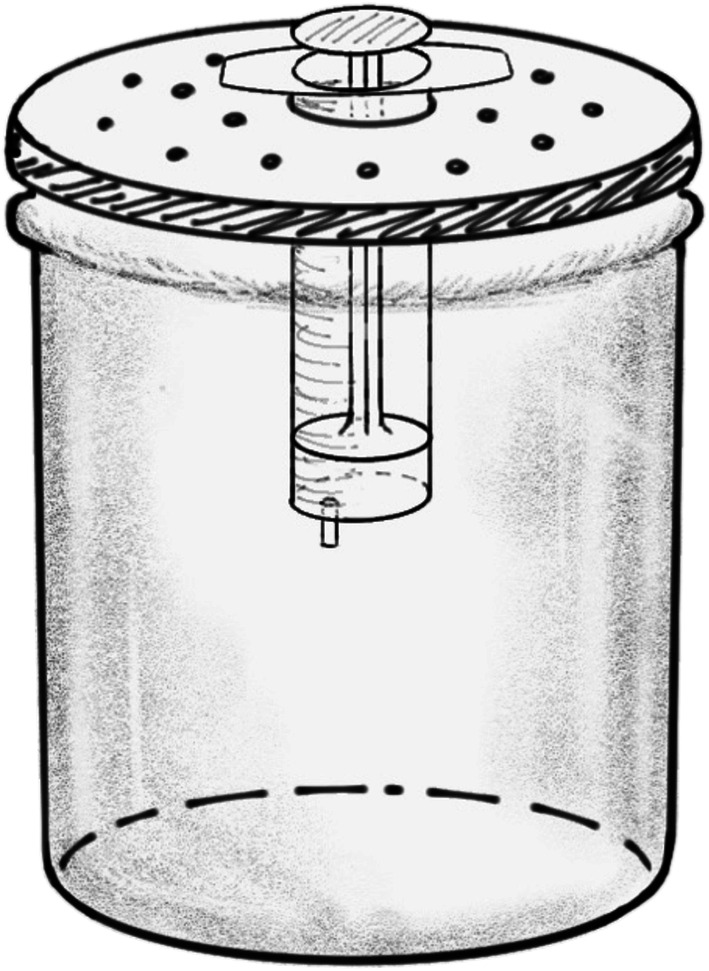
Screw top glass as used for the experiment; the lid is provided with air holes and a hole for a 10 ml syringe

Starting at day 10 after oviposition, the containers were checked daily for pupae. Day 10 was chosen because it marks the middle point between the beginning of the migration phase and the hatching of the first flies at 25 °C, which was the experimental temperature that resulted in the fastest development. The pupae were transferred into screw top glasses, the lids of which were also provided with air holes. Access for a 10 ml syringe was provided in the middle of the lid (Fig.1). This hole allowed for the supply of water, food or the relocation of specimens, while minimizing the risk of adult escape.

Pupae were checked every 24 h for fly eclosion and hatched flies were separated on a daily basis to minimize age variations within a study group. Groups of 25–30 flies were kept in the dark, at two constant temperatures each (16° or 25 °C) with sugar and water ad libitum. Samples of 5–10 flies were removed at the age of 0 (< 24 h), 5, 10, 15, 20 and 25 days respectively and killed by freezing at −20 °C. These specimens were then stored at the same temperature. By repeating this procedure three times, a total of about 25 flies per sex, age and temperature were obtained, aiming for a balanced number between the sexes.

### Processing and measurement

The fly head was separated from the body and transferred into a 1.5 ml micro tube. It was ground with a pestle in 500 μl of 0.05 M Tris-HCl-Buffer (pH 8.0) to facilitate the dissolution of the water soluble pteridines in buffer. Tubes were centrifuged for 5 min at 6000 g, then 150 μl of the supernatant was transferred into a well of a 96-well microplate. The measurement of the pteridine concentration was modified according to Zhu et al. [18], using fluorescence intensity after stimulation. The pteridine fluorescence was measured in a microplate reader (Tecan, Infinite M200) at an emission wavelength of 450 nm after excitation with 360 nm. The intensity of the emitted light is given in relative fluorescence units (RFU). As a reference for adult fly size, the length of the posterior cross vein [32, 33] of the right wing, and the head width (widest point), were measured on an image captured with an AxioCamICc1 through a binocular microscope. Axio Vision 4.7.1 (08/2008) was used for measurements and graph paper was used as a size reference for calibration. Pteridine RFU values were then plotted against fly size and sex.

Heads and pteridine solutions were stored and processed in a frozen or cooled state at any time, except during measurement of the fluorescence which was being performed at room temperature. The samples were protected from direct light by aluminum foil.

### Accumulated degree days (ADD)

In order to quantify ageing across temperatures we use the concept of accumulated degree days (ADD). ADD measures the accumulated thermal energy given to an insect as temperature over time (days) and serves as a parameter of physiological age, mostly independent of the temperature conditions during rearing [5].

The formula for summing the accumulated temperature is as follows:

$$ \mathrm{ADD}=\left(\mathrm{T}-{\mathrm{T}}_0\right)\ \mathrm{x}\ \mathrm{age}\ \mathrm{in}\ \mathrm{days} $$

T: mean ambient temperature.

T_0_: lower threshold temperature 9 °C [5, 34–36].

### Statistical analysis

Pteridine values of the different repetitions were examined according to their sex for differences between the groups using a t-test. Pooling of repeated groups only took place if no significant difference (*p* > 0.05) could be detected. The examination of the minimum and maximum values for outliers was carried out with the Dean Dixon test after confirmation of the normal distribution (Kolmogorov-Smirnov test).

A correlation between head width or length of the posterior cross vein and pteridine RFU value was analyzed using Pearson’s correlation coefficient. The calculations were carried out with Excel.

### Mock case

In order to test the applicability of the method for age determination of flies in real cases, a mock case with 5 flies per age group and 3 age groups per temperature (16 °C and 25 °C) was set up. The true age of the fly was unknown to the person carrying out the test. The measured pteridine level was compared with that of the groups of the known age (same sex) and a minimum age of the fly was determined using the generated reference data.

## Results

**Fig. 2:**
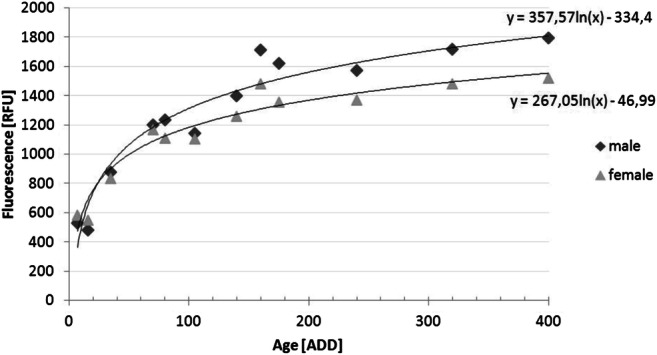
Mean fluorescence values separated by sex for the respective ADD (*n* = 16–25 per sampling, both rearing temperatures combined)

**Fig. 3:**
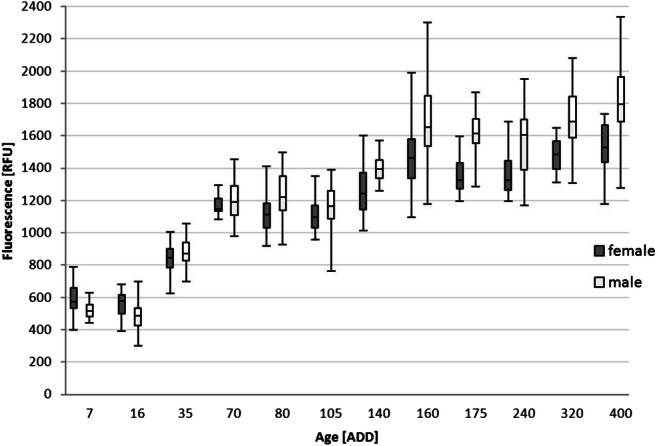
Box plots of the fluorescence values separated by sex for the respective ADD (n = 16–25 each per sampling, both rearing temperatures combined) where the dividing line of the box indicates the median and the end of the whiskers the maximum and minimum value; outliers are excluded

**Fig. 4:**
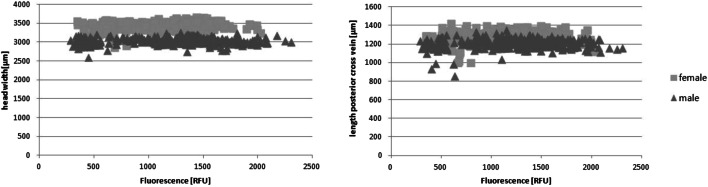
Correlation of fluorescence values and head width (r = −1.6 × 10^−9^) or fly size (r = −1.4 × 10^−9^)

Fluorescence level and thus the extracted pteridine from the heads of *L. sericata* showed a logarithmic increase with rising ADD (Fig. 2). The comparison of flies <70 ADD with flies >70 ADD showed a significant difference between these groups, as did the comparison of flies with an age of <5 days, 5–10 days and > 10 days within one temperature. Next to age (*p* < 0.05), the sex of the fly showed a significant influence (*p* < 4.09 × 10^−6^) on pteridine levels (Figs. 2 and 3). Male flies achieved higher fluorescence values than females, while they were smaller in size. No significant relationship between size (r = −1.4 × 10^−9^) of the fly and the measured pteridine level could be determined (Fig. 4).

**Fig. 5:**
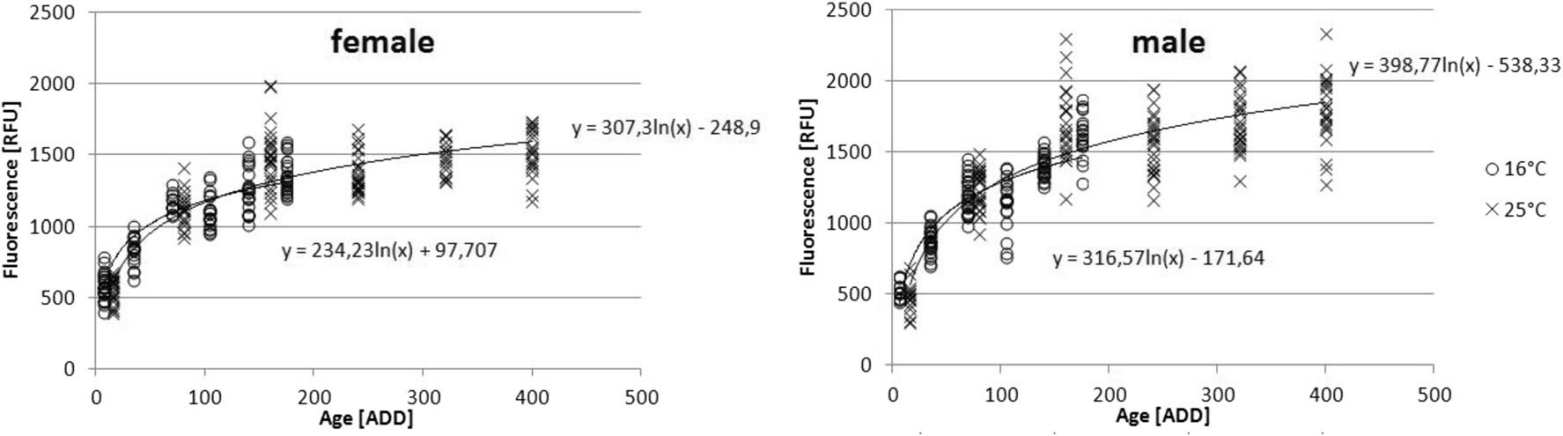
Fluorescence values separated by sex and temperature

Based on the arithmetic mean of each group of the same age and sex (*n* = 16–25), the increase of pteridine can be described by the following formulas: y = 267.05ln(x) – 46.988 (female) and y = 357.57ln(x) - 334.4 (male, Fig. 2), considering the two rearing temperatures together. Despite the fact that pteridine values are considered to be temperature-dependent [17–19], both applied temperatures showed similar approximate curves after transforming age in ADD (Fig. 5).

The comparison of the estimated minimum age (based on the fluorescence level) with the true age of the flies in the mock case demonstrated that an overestimation of the real age in ADD only occurred in two out of 30 samples (sample 4 and 5 of group A, age was <24 h each) (Table 1). However, with increasing age, there was an increasing discrepancy between the true and estimated age, while the minimum age should always be below the real age.

**Table 1:** Comparison of the estimated minimum age (based on the fluorescence level) in the mock case with the real age of the fly

Group	Sample	Temperature	Fluorescence value	Sex	Age		estimated minimum Age	
					[d]	[ADD]	[d]	[ADD]
A	1	16	380	f	≤1	7	≤1	7
	2	16	420	f	≤1	7	≤1	7
	3	16	397	f	≤1	7	≤1	7
	4	16	355	m	≤1	7	≤1	16
	5	16	333	m	≤1	7	≤1	16
B	1	16	749	f	10	70	≤1	7
	2	16	686	f	10	70	≤1	7
	3	16	764	m	10	70	5	35
	4	16	712	m	10	70	5	35
	5	16	713	m	10	70	5	35
C	1	16	864	f	20	140	5	35
	2	16	919	f	20	140	5	35
	3	16	1032	m	20	140	5	35
	4	16	781	m	20	140	5	35
	5	16	843	m	20	140	5	35
D	1	25	1335	f	10	160	5	80
	2	25	997	m	10	160	5	35
	3	25	1034	m	10	160	5	35
	4	25	1213	m	10	160	5	70
	5	25	1297	m	10	160	5	70
E	1	25	1339	f	15	240	5	80
	2	25	1398	f	15	240	5	80
	3	25	1237	f	15	240	5	70
	4	25	1450	f	15	240	10	140
	5	25	1027	m	15	240	5	35
F	1	25	1424	f	25	400	10	140
	2	25	1121	f	25	400	5	70
	3	25	1022	f	25	400	5	80
	4	25	1727	m	25	400	10	160
	5	25	1655	m	25	400	10	160

## Discussion

Until now, forensic entomology ran the risk that the calculated PMI_min_, although correct, would be significantly different to the actual time since death when empty puparia were present as it is unclear when the adult fly left the puparium; it could be hours, days, weeks or months prior collection of the specimens (see [37] for discussion about ageing the chemical degradation of empty puparia). By assuming that the fly hatched immediately before the puparium was found, the PMI_min_ correctly takes this possible delay into account. Nevertheless, it is still possible that there is a loss of information in this scenario. Hence, for estimating the age of adult flies a parameter like the pteridin level in the fly’s eye could be useful.

A positive correlation between pteridine levels and age has been detected in most insects that have been studied, including many adult Diptera, with differences just in the pattern of increase depending on the species. While the flies *Musca domestica* [17], *Chrysomya bezziana* [19], *Calliphora vicina* [8], *Chrysomya megacephala, Cochliomyia macellaria* and *Phormia regina* [27] (with the exception of *P. regina* reared at 5.4 °C showing a linear increase), *Anastrepha ludens* [38] as well as *Lucilia sericata* [10] show a curvilinear increase of pteridine with age, the Diptera *Chrysomya megacephala* [18], *Boettcherisca peregrina* [6] and *Glossina* spp. [20] show a linear increase. In other insects such as the honey bee *Apis mellifera* [39] a correlation between ageing and pteridine level was also found, while, for example, the ants *Polyrhachis sexspinosa* [40] and *Platythyrea punctata* [41] showed no direct correlation.

Pteridine is currently attributed three main tasks in relation to insects: To act as a filter for UV light [25, 26], as pigmentation, and as a form of nitrite excretion [42]. Assuming that in the eye of the fly its function as a filter is predominant would explain the logarithmic increase due to an adjustment of the pteridine synthesis as soon as the concentration necessary for protection has been reached. If the increase in pteridine is mainly due to the excretion of nitrates [22], a linear increase would be more likely based on the assumption of a continuous production. As diet [10, 17, 19], light intensity and duration [26] as well as movement [17] have no influence on the pteridine value, which would have to be the case if pteridines were a pure degradation or excretion product and thus had to correlate with activity and metabolic rate, the assumption that pteridines are simply excretion products should be rejected. Furthermore, no correlation of fly size, indicated by vein length or head width, on the pteridine value could be demonstrated (Fig. 4). This finding contradicts previous observations concerning *L. sericata*, showing a significant correlation of fly size and pteridine level [10]. Further information on influencing factors could maybe be provided by testing pupae and larvae; the latter has shown a linear increase of pteridine with larval age in blow flies *Ch. megacephala* and *Ch. rufifacies* [43].

The present study confirmed a positive curvilinear relationship between the age of the examined fly species and the measured fluorescence value, i.e. the pteridine-level as mentioned before by Wall et al. [10] for *L. sericata*. After 70–80 ADD the increase slows down and the curve approaches a maximum value and some sort of saturation. Hence, age determination of *L. sericata* on the basis of pteridine levels seems to be limited to a physiological age of about 70 ADD. For older specimens, only the indication of a minimum age of about >70 ADD is possible. The mock case also confirmed this limitation of age determination up to 70–80 ADD of the adult fly. Nevertheless, by using the fluorescence value it was possible to determine a minimum age, which was never above the actual age. Male flies, which can be easily recognized by their holoptic eyes, overall exhibited a higher pteridine level than female flies. This phenomenon has been observed in other fly species before [17, 19, 26], but as yet no explanation for this has been provided. For forensic purposes this must be taken into account; the sex of the fly determines which of the two developed formulas must be used for age calculation.

Pteridine approached a stable level at around 140–160 ADD (Fig. 5) after eclosion of the adult fly, which coincides with previous observations in *Calliphora vicina* (there: *C. erythrocephala*) [26] and restricts the use of quantitative pteridine fluorescence for age determination of adult flies to this period, which covers approximately half the life of a fly [44]. Nevertheless, even such a short and restricted period of time would already mean an improvement for the PMI_min_ estimation, since a wider range than just the time taken by juvenile development and metamorphosis could be covered by adding the age of the adult fly.

Pteridine fluorescence analysis of dead flies collected at the site is another possibility that could be explored in the future as there is a clear gap of knowledge when it comes to pteridine stability and half-life, for example due to photosensitivity. Even though pteridine is considered photosensitive in vitro, it was stable in vivo [21, 26], which applied not only to living animals but also to freeze-dried heads, making it easy to store and meaning immediate processing was not required.

Comparing the two temperatures (16 °C and 25°) with their respective ADD (Fig. 5), an almost congruent pattern can be seen in the common course (illustrated by the approximation). In order to compare the patterns over its entire length it would be necessary to collect samples of older flies at 16 °C to add higher ADD values for this temperature. In addition, it seems advisable to analyze more temperature profiles to get a more accurate picture of the fluorescence progression, especially within the relevant range up to 70 ADD. Nevertheless, the logarithmic shape can be seen clearly in both curves as well as their associated maximum value of pteridine. The scattering of the fluorescence values within a temperature group results in an increasing overlap of ranges with increasing ADD, making it impossible to clearly assign a fluorescence value to a distinct age. But considering the rising mortality of flies with increasing ADD [45], the lower ADD ranges also seem to be the more relevant for everyday application.

Last but not least it should be mentioned that even if the age of the adult fly is successfully estimated, its previous development on the body must be ensured. It could have developed on site or it could have arrived as imago from outside. This problem restricts the use of this method to indoor cases. If there is a large number of flies, belonging to the same age cohort and the corresponding species is represented by several empty puparia at the place of discovery, it is likely that they have developed on site [8]. To estimate whether a fly has developed on site or may just have entered from outside, for example 1 day before sampling, wing fray analysis and determination of sex ratios could also provide an indication of the origin of present flies, where a greater amount of wing damage as well as a female dominance hint at flies that entered from outdoors [46]. This indicates that the combination of different methods could help to improve the interpretation of entomological evidence. In the future, stable isotope analysis might also prove to be a useful tool for associating flies with a food source [47].

Altogether, the quantitative pteridine fluorescence provides a good approach to age determination of adult *L. sericata* within the first days after hatching. Apart from age and sex, no further factors seem to influence the fluorescence value, which is an advantage compared to other methods.

### Key points

1. The pigment pteridine accumulates in the eyes of Lucilia sericata with increasing age and can be measured by fluorescence spectroscopy.

2. The pteridine level allows conclusions regarding the minimum age of the fly, which can be used to narrow down the post-mortem interval.

3. The concept of accumulated degree days (ADD) was used to quantify ageing across temperatures.

4. While sex and age showed significant influences on the fluorescence value and therefore on the pteridine level, fly size did not.
